# Evaluating Association of Maternal Nutritional Status With Neonatal Birth Weight in Term Pregnancies: A Cross-Sectional Study With Unexpected Outcomes

**DOI:** 10.7759/cureus.17621

**Published:** 2021-08-31

**Authors:** Rumana Sangi, Aliya K Ahsan, Asma T Khan, Syed Nurul Aziz, Meher Afroze, Saifullah Jamro, Tayyaba Haque, Zain Ali Zaidi, Sameer S Tebha

**Affiliations:** 1 Pediatrics, Chandka Medical College, Shaheed Mohtarma Benazir Bhutto Medical University (SMBBMU), Larkana, PAK; 2 Pediatrics, Abbasi Shaheed Hospital, Karachi, PAK; 3 Internal Medicine, Larkin Community Hospital, South Miami, USA; 4 Pediatrics, Larkin Community Hospital, Miami, USA; 5 Pediatrics, Jinnah Medical and Dental College, Karachi, PAK; 6 Pediatrics, Shaheed Mohtarma Benazir Bhutto Medical University (SMBBMU), Larkana, PAK; 7 Medicine, Jinnah Medical and Dental College, Karachi, PAK; 8 Neurology and Neurosurgery, Jinnah Medical and Dental College, Karachi, PAK; 9 Clinical and Translational Research, Larkin Community Hospital, Miami, USA

**Keywords:** cross-sectional study, low birth weight, maternal malnutrition, mid-upper arm circumference, nutritional status

## Abstract

Introduction

Birth weight is described as the primary determinant of the chances of survival among newborns. Low birth weight (LBW) is considered to be a major public health issue, especially among developing countries where poor maternal nutritional status is identified as a cause of both long and short-term adverse consequences. In developing countries, the majority of the LBW infants are born at term but are affected by intrauterine growth restriction, which might have begun early in the pregnancy period. We conducted this study in order to determine the possible effects of the poor nutritional status of mothers on the birth weight of their newborns. However, in disparity to the previous literature, our study evaluated unpredictable results.

Methods

This is a cross-sectional study that was conducted at two tertiary care teaching hospitals from November 2020 to April 2021 in order to determine the possible effects of the poor nutritional status of mothers on the birth weight of their newborns. 156 women both primigravida and multigravida of ages between 15 and 50 years and those who delivered low birth weight (LBW) babies of either gender at term (37-40 weeks of gestation) were included. For all mothers who delivered LBW (<2500 g) at term, their mid-upper arm circumference (MUAC) was measured by inelastic tape. Mothers with MUAC less than 21 cm were considered malnourished.

Results

A total of 156 study participants were included in the study, with majority (n=112, %=71.8%) of them between the ages of 20 and 30 years. The mean age of all included participants was calculated to be 25.96±4.54 years (ranging from 18 to 38 years). Prevalence of maternal malnutrition was observed in 41 (26.3%) of the included women who delivered LBW babies, in contrast to high prevalence rates in previous literature.

Conclusions

In contrast to the previous literature, our study has shown that the nutritional status of mothers has no significant impact on the weight of neonates and the majority of neonates in our study were not severely low weight

## Introduction

Birth weight is described as the primary determinant of the chances of survival among newborns. Low birth weight (LBW) is considered to be a major public health issue, especially among developing countries where poor maternal nutritional status is identified as a cause of both long and short-term adverse consequences [1,2]. The LBW is described as birth weight below 2500 grams regardless of gestational age [3]. In developing countries, the majority of the LBW infants are born at term but are affected by intrauterine growth restriction, which might have begun early in the pregnancy period [4].

The LBW is an indirect measure of intrauterine malnutrition and is considered to be a very important risk factor for fetal as well as neonatal morbidity and mortality [1,2]. It is estimated that more than half of the newborns in developing countries are not weighed, which is a big concern considering the global incidence of LBW [5]. As per UNICEF, around 96% of LBW newborns belong to developing nations while 72% belong to South Asia. Physical well-being and cognitive development are also impacted by LBW [6,7]. Local data suggested the prevalence of LBW to be between 5% and 23% [8,9]. Maternal nutritional status is directly related to the quality of diet, educational status, and income [10]. Mid-upper arm circumference (MUAC) is an uncomplicated, effortless but still reliable method of assessing maternal nutritional status which is considered credible enough to be used as a single anthropometric measurement. Previous studies have also used this method to evaluate the maternal malnutrition and its association with birth weight [11,12]. A study conducted in India suggested that MUAC and birth weight measurement can substitute other complicated methods of evaluating maternal malnourishment in lower-income or underdeveloped countries [13]. Researchers have credited about 50% of LBW cases to maternal nutritional factors both prior to and during the pregnancy [14].

As local data is deficient on LBW, especially with regard to maternal malnutrition measured by maternal mid-upper arm circumference (MUAC), this research was aimed to determine the effect of the nutritional status of a mother over the birth weight of the newborn. If it seems to affect the birth weight, then policies can be made to maintain the nutritional status of mothers hence decreasing the number of newborns with low birth weight. However, in disparity to the previous literature, our study revealed unpredictable results.

## Materials and methods

This is a cross-sectional study conducted at pediatric departments of two tertiary care teaching hospitals in Larkana, Pakistan, from November 2020 to April 2021. Approval from the institutional ethical committee was acquired. Each participant was included in the study after a written informed consent. A sample size of 156 women was calculated considering the prevalence of maternal malnutrition as 11.5%, with a confidence interval of 95% and a margin of error of 5%.

As per non-probability consecutive sampling, 156 mothers (both primigravida and multigravida) aged 15-50 years who delivered LBW babies of either gender at term were included. Mothers delivering babies with any congenital anomaly and mothers having twin pregnancies or those with any chronic illness like diabetes, hypertension, cardiac disease were not enrolled. Women having pre-eclampsia, eclampsia, or those having hepatitis B and C were also excluded.

All pregnant women and newborns from the neonatology ward of the pediatric department and postnatal wards that met our inclusion criteria of mothers aged between 15-50 years who delivered LBW babies of either gender at term were enrolled in this study. All mothers who delivered LBW (<2500g) at term, their mid-upper arm circumference was measured by inelastic tape. Mothers with MUAC less than 21cm were considered malnourished.

Data was analyzed on SPSS version 26.0 (IBM Corp., Armonk, NY). Percentage and frequency were calculated for categorical variables like the gender of the baby, parity, residence, education level, booking status, and maternal nutritional status. Mean and standard deviation was calculated for quantitative variables like age of mother, gestational age, and economic status, and Pearson correlation was done to access the correlation of MUAC with LBW. Stratification was done further to control effect modifiers like age of mother, educational status, gestational age, educational status, economic status, duration of pregnancy to see the effect of these on outcome variables taken, applying Chi-square test considering p-value < 0.05 as significant.

## Results

A total of 156 study participants were included in the study, with 112 (71.8%) of them between the ages of 20 to 30 years. The mean age was calculated to be 25.96±4.54 years (ranging from 18 to 38 years). Mean gestational age was calculated to be 37.82+0.96 weeks (ranging from 37 to 40 weeks). There were 31(19.87%) women who were primigravida. Most of the women, 120 (76.9%) were illiterate. The gender of the newborn baby was male in 88 (56.4%) cases. Characteristics of study participants are shown in (see Table 1).

**Table 1:** Basic characteristics of the included study participants. Note that the majority of participants were between ages 20-30 years, multigravida women from rural areas with low literacy rates. Majority of the newborns were males with an average weight of 2.16 kg followed by females weighing an average of about 2.143 kg.

Characteristics		Value (% or mean)
Age (years)	<20	20 (12.8%)
	20-30	112 (71.8%)
	>30	24 (15.4%)
Parity status	Primigravida	31 (19.9%)
	Multigravida	125 (80.1%)
Residential status	Urban	50 (32.1%)
	Rural	106 (67.9%)
Booking status	Booked	100 (64.1%)
	Un-booked	56 (35.9%)
Newborn gender	Male	88 (56.4%)
	Female	68 (43.6%)
Average weight of newborns	Male	2.160 (mean value)
	Female	2.143 (mean value)

Stratification analysis of study variables was performed with respect to the prevalence of maternal malnutrition. Factors such as age, educational status, gestational age, household income, parity status, residential status, booking status, LBW, and newborn gender were analyzed. Table 2 shows the comparison of maternal characteristics with respect to nutritional status. However, no statistical significance was found (p > 0.05).

**Table 2:** Comparison of characteristics of mothers who delivered LBW with respect to nutritional status (n = 156). Note that no significant correlation of maternal malnourishment with any evaluated variable was found. LBW: low birth weight.

Characteristics		Maternal malnutrition		p-value
		Yes (n = 41)	No (n = 115)	
Age group (years)	≤ 20 years	9(22.0%)	11(9.6%)	0.119
	21 to 30 years	27(65.8%)	85(73.9%)	
	>30 years	5(12.2%)	19(16.5%)	
Educational status	Illiterate	34(82.9%)	86(74.8%)	0.490
	Primary	4(9.8%)	14(12.2%)	
	Middle	1(2.4%)	3(2.6%)	
	Matric	2(4.9%)	4(3.5%)	
	Intermediate	0(0%)	8(7.0%)	
Gestational age (weeks)	37-38	31(75.6%)	94(81.7%)	0.398
	39-40	10(24.4%)	21(18.3%)	
Household income (PKR)	≤ 15,000	31(75.6%)	86(74.8%)	0.490
	15,000 to 35,000	10(24.4%)	27(23.5%)	
	>35,000	0(0%)	2(1.7%)	
Parity status	Primigravida	9(22.0%)	22(19.1%)	0.698
	Multigravida	32(78.0%)	93(80.9%)	
Residential status	Rural	31 (75.6%)	75 (65.2%)	0.221
	Urban	10 (24.4%)	40 (34.8%)	
Booking status	Booked	27(65.9%)	73(63.5%)	0.785
	Un-Booked	14(34.1%)	42(36.5%)	
Newborn gender	Male	28 (68.3%)	60 (52.2%)	0.074

Pearson correlation test was applied in order to evaluate the association and impact of maternal malnourishment on the birth weight of newborns. A low significance value of 0.145 was attained, showing only mild correlation, as shown in Table 3.

**Table 3:** Correlation of maternal mid-upper arm circumference and birth weight of newborns. Note that the significance of 0.145 representing the mild association of lower maternal MUAC with decreased birth weights of the newborns. MUAC: mid-upper arm circumference.

		Mid-upper arm circumference	Birth weight
Mid-upper arm circumference	Pearson correlation	1	0.117
	Significance (two-tailed)		0.145
Birth weight	Pearson correlation	0.177	1
	Significance (two-tailed)	0.145	

## Discussion

Although LBW is prevalent in many parts of the world, particularly in developing countries, South-Asia is the highest contributor to LBW newborns as WHO and UNICEF in 2011 estimated around 27% of all live births from South-Asia to be LBW babies [15].

Women of reproductive age are more prone to nutritional insufficiencies, especially those from rural areas, as improper food intake, inadequate quality of diet, recurrent infections, and less duration of the inter-pregnancy interval are some of the most important contributors of maternal as well as newborn malnutrition [16-20]. Further, maternal anthropometry and her nutritional intake diversity before and during pregnancy are critical factors influencing birth weight, but in our study, none of these factors were significant contributors towards neonatal weight.

In our study, there were 67.9% women from rural areas, while 35.9% cases were un-booked. This could be because of a lack of general and obstetrical healthcare awareness in society. Our healthcare setting provides access to most nearby villages while people living in rural areas lack awareness about health measures and health facilities. People are poor and have large family members. They also don't believe in antenatal care and perceive birth to be a completely natural process. They bring their women to healthcare facilities, specifically when they feel seriously ill, and keep insisting on vaginal birth. In our study, most of the women were illiterate (76.9%) while 75% of the women had low household incomes below PKR 15,000 (158 PKR=1 USD), but none of these factors proved to be statistically significant factors in our study.

Lack of education and dietary knowledge could be contributing factors to maternal nutrition, as has been proved in some studies [21]. Furthermore, emphasis should also be given on the availability and accessibility of diverse food items and to promote healthy diets and healthy lifestyles through nutrition counseling, especially as the nutritional needs of pregnant women increase during pregnancy.

In our study, the prevalence of maternal malnutrition among women who delivered LBW babies was observed in 26.3%. Girma S et al. from Ethiopia, evaluating factors linked with LBW, noted that 52.7% of mothers of LBW newborns were having malnutrition (MUAC < 23 cm) in comparison to 13.4% among controls (p=0.001) [22]. They also concluded that lack of proper dietary habits and nutritional support was having a significant association with LBW, but our study disproves this hypothesis as in our study, there was no significant association between maternal malnutrition and LBW babies. Gebremedhin M et al. also revealed that newborns as LBW were having maternal malnutrition (MUAC<21 cm)among 44.5% mothers in comparison to 33.8% among controls (OR 1.56, 95% CI: 0.82-2.97) but yet again in our study the same variables disprove this hypothesis [23]. Local data has also revealed the presence of maternal anemia to contribute to LBW among newborns, this could have been a factor, but in our study, we didn't evaluate anemia [24]. During pregnancy, women should be counseled about a proper and healthy diet. Weight gain before and during pregnancy needs to be monitored to enhance chances of good outcomes in mothers newborns, but more importantly, our study suggests that other factors that could contribute towards LBW in neonates should be thoroughly evaluated as well, and only maternal nutritional status shouldn't be taken as an outcome measure.

Our study had some limitations. We had a comparatively small sample size which could have reduced the amount of statistical power to identify meaningful associations with study variables. The dietary intake information in retrospective surveys is always prone to reporting bias. Also, the dietary information in the last one month may not accurately represent a dietary pattern of the entire duration of pregnancy. We were also unable to record the influence of maternal anemia, usage of folic acid and calcium supplements during the pregnancy period.

## Conclusions

Most studies have proven that maternal malnutrition directly impacts and influence the weight of neonates, but our study has proven that the nutritional status of mothers has no significant impact on the weight of neonates and the majority of neonates in our study were not severely low weight, and as such we have hypothesized that there are further factors that could lead to LBW in neonates except maternal nutrition. Moreover, all the demographic factors that we analyzed have also proven no association between gender, age, or gestational status of mothers, nor were educational status or booking status; thus, more thorough analyses of specific nutrients should be conducted, and larger cohort specifically prospective studies should be conducted to validate our findings.
